# Beyond Species Averages: Intraspecific Trait Variation Reveals Functional Convergence Under Invasion

**DOI:** 10.3390/insects16111094

**Published:** 2025-10-24

**Authors:** Zhixing Lu, Xinyu Wang, Xiang Zhang, Youqing Chen

**Affiliations:** 1 Institute of Highland Forest Science, Chinese Academy of Forestry, Kunming 650224, China; 2 Yunnan Key Laboratory of Breeding and Utilization of Resource Insects, Kunming 650224, China; 3 Key Laboratory of Protection and Utilization of Insects, National Forestry and Grassland Administration, Kunming 650224, China

**Keywords:** biological invasion, community assembly, competitive filtering, traits, intraspecific trait variation (ITV), *Solenopsis invicta*

## Abstract

**Simple Summary:**

We studied ant communities along a gradient of invasion by *Solenopsis invicta* in Yunnan, China. Extreme competition from the invader filtered native species into a narrow functional zone, producing community-level functional convergence. Intraspecific trait variation enabled rapid trait shifts that aided persistence, and the invader exhibited density-dependent phenotypic plasticity. By linking individual variation to community patterns, we clarify when niche differentiation yields convergence and offer guidance for predicting and managing responses under severe biotic pressure.

**Abstract:**

Biological invasions provide a unique window into community assembly. While classic theory predicts that native species must differentiate their niches to coexist with an invader, the actual outcomes under intense pressure are complex. Our study examines community reassembly under extreme pressure from the invasive ant *Solenopsis invicta*. We found that while native species do differentiate themselves from the invader, the overwhelming competition constrains this process, forcing survivors into a narrow, shared functional space. This constrained niche differentiation produces a pattern of community-level functional convergence, a process where functionally dissimilar communities become more similar under intense environmental filtering, as survivors are forced into a narrow, shared niche space. The capacity for these rapid, adaptive niche shifts is rooted in intraspecific trait variation (ITV). We also identified a dynamic feedback loop through density-dependent phenotypic plasticity in the invader. By showing how the foundational process of niche differentiation leads to a convergent outcome under extreme pressure, our work clarifies the rules of community assembly in an increasingly invaded world.

## 1. Introduction

The assembly of ecological communities is a central theme in ecology. Contemporary theory synthesizes this process into four fundamental mechanisms: selection, drift, dispersal, and speciation [1,2,3]. Within this framework, biotic interactions such as competition form a key component of selection, where classic niche theory predicts that coexisting species must occupy distinct niches to minimize competitive pressure [4,5]. This principle, often termed limiting similarity, predicts that the intense biotic pressure from an invader acts as a strong environmental filter, excluding native species with overlapping functional traits [6,7,8]. However, this framework is often tested in systems with moderate competition, leaving its predictions under-explored in communities facing extreme pressure from a single, overwhelmingly dominant invader.

Biological invasions therefore offer a unique opportunity to test and refine niche theory. The fire ant, *Solenopsis invicta*, is a notoriously successful invader, known for its aggressive behavior and ability to achieve high population densities, which can devastate native biodiversity [9,10,11]. Its invasion creates a steep gradient in biotic pressure [12,13,14,15], providing a natural experiment to observe community reassembly in real-time [16,17,18]. The extreme competition imposed by *S. invicta* allows us to ask a more specific question: when niche space is severely constrained by a dominant competitor, does the classic pattern of niche differentiation still hold, or do alternative assembly rules emerge? Its establishment creates intense competitive pressure that should promote strong selective filtering, making it possible to detect functional re-sorting if it occurs [19,20]. Recent trait-based studies have begun to reveal the mechanisms underlying *S. invicta*’s invasion success, indicating that its establishment relies on a combination of niche differentiation and competitive superiority across multiple functional dimensions [8,21,22].

Furthermore, most studies on community assembly rely on species-mean trait values, which implicitly assumes that species are phenotypically uniform [23,24,25,26]. This approach overlooks intraspecific trait variation (ITV), the phenotypic differences among individuals of the same species [21,26]. Recent studies highlight that ITV is a crucial source of adaptive potential, allowing populations to rapidly respond to new selective pressures and significantly impacting higher-order ecological processes such as community assembly and invasion success [24,27,28,29,30]. In the context of biological invasions, ITV may enable native species to shift their niche away from an invader, facilitating persistence in ways that species-level analyses cannot detect [23,24,28]. Ignoring ITV may therefore lead to an incomplete, or even misleading, understanding of the mechanisms governing community reassembly under invasion [23,31].

In this study, we investigated the functional trait structure of ant communities along a natural invasion gradient of *S. invicta*. We aimed to understand how an extreme biotic filter reshapes community assembly. We specifically tested whether the intense competition from *S. invicta* leads to a simple divergence of native species into available niche space, or if it results in more complex, community-level patterns. By analyzing traits at the individual level, we also quantified the role of intraspecific trait variation in this process. We specifically ask: (1) Does the invasion of *S. invicta* lead to functional homogenization or functional re-sorting of the native ant community? (2) What is the role of intraspecific trait variation (ITV) in mediating the persistence of native species in the presence of the invader? (3) Does the invader itself exhibit trait plasticity across different invasion contexts? By addressing these questions, we aim to provide a more nuanced understanding of community reassembly under the pressure of a dominant invader, highlighting the dynamic interplay between interspecific competition, niche partitioning, and intraspecific adaptation.

## 2. Materials and Methods

### 2.1. Study Areas

The study was carried out in Kunming City, Yunnan Province, China, which has a subtropical plateau monsoon climate. The region receives about 992 mm of rainfall annually and has an average temperature of 15.2 °C. Over the past thirty years, the warmest month averaged 20.1 °C, while the coldest averaged 8.4 °C (ClimateAP v3.01 [32] normal data 1991–2020; N 25.0367, E 102.7914). From November to February, temperatures in Kunming are unusually low for optimal red fire ant activity.

We established the three habitat types (SF/EC/SI) at three primary locations in Shalang in Kunming, Yunnan Province, with distances exceeding 1 km between each location. In total, our design comprised nine plots (3 locations × 3 habitats). Each location contained one plot per habitat type. We chose three distinct habitat types along a clear invasion gradient: (1) Secondary Forest (SF), located within secondary forests mainly composed of *Pinus yunnanensis* and *Albizia julibrissin*, with a closed canopy covering 50–70%. No *S. invicta* mounds or workers were observed in these habitats during the entire study. (2) Forest Edge (EC): These less-invaded sites were situated in open, sparsely vegetated zones with canopy cover of 20–35%, immediately next to secondary forests. They exhibited significantly lower mound density and forager activity of *S. invicta* compared to SI areas. Nevertheless, these regions still supported a relatively abundant and diverse native ant community. (3) Open Habitat (SI) sites were found in areas with sparse vegetation, about 15% cover, that had been colonized by *S. invicta* for approximately three to five years, featuring high population densities and numerous mature mounds, indicating an established invasion.

### 2.2. Ant Sampling

To capture the distinct seasonal variations in the region, ant communities were surveyed at two critical time points in 2021. The first survey was conducted in early June, representing the end of the pronounced dry season in Yunnan. The second survey took place in late August, which corresponds to the peak of the monsoon, or rainy season. We used pitfall traps, a standardized technique that yields quantitative measures of surface-active ant abundance and diversity. This method is especially useful for examining competitive interactions with ground-foraging invasive species such as *S. invicta*, as it accurately detects the foraging worker caste, which is the main point of interspecific competition. In each plot, 15 pitfall traps were placed as a line, spaced 10 m apart. These traps, made of plastic cups measuring 8 cm in diameter and 15 cm in height, were filled with 50 mL of a 50% ethylene glycol solution to preserve the specimens. The traps remained active for 48 h in the field. This resulted in a total sampling effort of 270 trap samples (habitats × 3 locations × 15 traps × 2 seasons). All ants captured were then transferred to 75% ethanol for preservation and later identification. Specimens were identified to the species level using China’s taxonomic keys and region-specific identification guides [33,34]. Those that could not be reliably identified were classified as morphospecies. Voucher specimens were stored at the Institute of Highland Forest Science, Chinese Academy of Forestry, Kunming, China.

### 2.3. Functional Trait Measurements

Following our standardized community sampling, we performed detailed morphological measurements to analyze functional diversity patterns. We measured five functional traits in worker caste individuals using a stereo microscope fitted with a TOUPCAM industrial digital camera and ToupView software (version 3.7). The five traits included head width (HW), which constrains access to microhabitat resources and correlates with bite force capacity [35,36]; mandible length (ML), a key determinant of prey handling ability and dietary niche breadth [37]; distance between eyes (DE), reflecting visual acuity and foraging efficiency [38,39]; femur length of the hind leg (FL), a proxy for locomotory performance and foraging range [35]; and Weber’s length (WL), a standard measure of overall body size that correlates with competitive dominance and resource monopolization ability [40]. To capture intraspecific trait variation (ITV), we measured all collected individuals of each species from each site rather than relying on species-average values.

### 2.4. Data Analysis

All statistical analyses were performed using R (version 4.1.2) and JASP (version 0.19.1) [41]. To examine differences in community composition, we performed ANOSIM tests using Bray–Curtis dissimilarity matrices calculated from square-root transformed abundance data. Community structure was visualized with Non-metric Multidimensional Scaling (NMDS), and analyses were carried out using PRIMER v7 [42]. Generalized linear mixed models (GLMM) were employed to examine variations in ant species richness and abundance across different plots. In these models, ‘plot’ was a fixed effect, while ‘survey month’ was included as a random factor to address possible temporal pseudoreplication. A Poisson distribution with a log link function was used, suitable for the count data. Pearson correlation analysis was conducted to examine the relationships between *S. invicta* abundance and native community metrics such as richness and abundance. To visualize the communities’ functional structure, kernel density plots were created for each trait within each habitat. Additionally, hierarchical trait-based analysis using the cati R package was performed to formally test the underlying assembly processes. This method calculates three T-statistics comparing observed community trait structures to null models: T_PC/PR_ (filtering from the regional to the local community), T_IC/IR_ (niche partitioning or competitive filtering within the community), and T_IP/IC_ (the contribution of ITV to community structure) [43]. The analysis results of network diagram, ridge plots and boxplots were generated using the CNSknowall platform (https://cnsknowall.com, accessed on 22 October 2025), a comprehensive web service for data analysis and visualization.

## 3. Results

### 3.1. Distinct Ant Community Structures Across the Invasion Gradient

**Figure 1:**
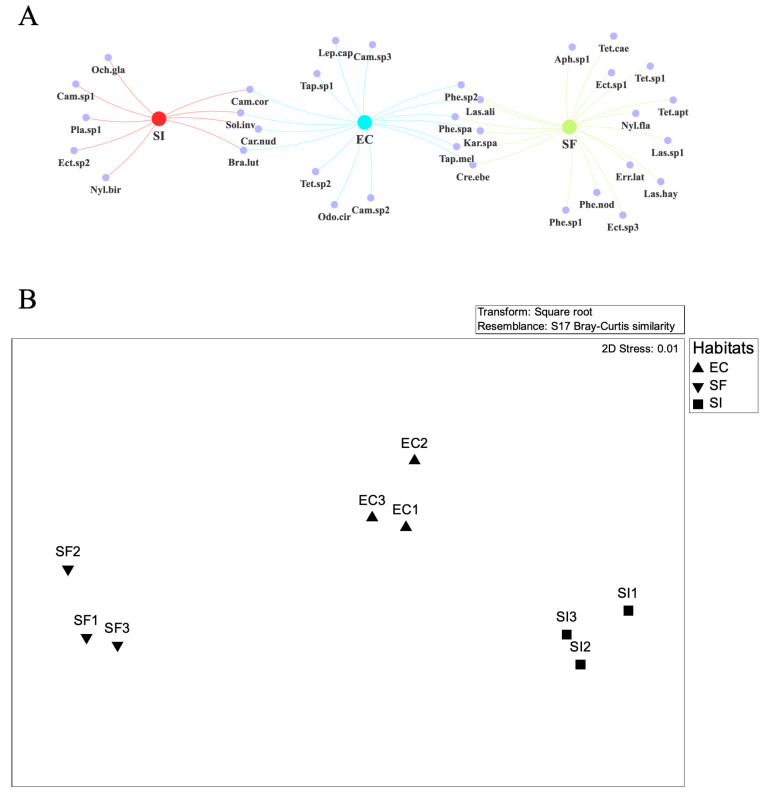
Illustrates the differences in ant community composition and structure across three habitat types. (**A**) A network diagram depicts the distribution of 28 ant species. The central nodes represent the secondary forest (SF), forest edge (EC), and *S. invicta*-invaded open habitat (SI). Peripheral nodes indicate individual ant species. Edges connect species to the habitats where they were collected. (**B**) Non-metric Multidimensional Scaling (nMDS) ordination displays the relationships among ant communities based on Bray–Curtis dissimilarity of square–root transformed abundance data. Each point corresponds to a sampling site (*n* = 3 per habitat).

We collected 2139 individual ants, representing 20 species from 17 genera across four subfamilies. The species–habitat network diagram revealed a highly structured distribution pattern across the three habitat types (Figure 1A). The secondary forest (SF) showed the greatest species richness with 15 species, 10 of which were unique to this habitat. In contrast, the *S. invicta*-invaded open habitat (SI) supported a distinct community of 9 species, characterized by the presence of the invader. The forest edge (EC) served as a less invaded zone, sharing species with both the secondary forest and the invaded areas. Non-metric multidimensional scaling (nMDS) ordination, which is based on species abundance, distinctly displayed three separate, non-overlapping clusters that exactly matched the three habitat types (stress = 0.01, see Figure 1B). These results clearly show a spatial separation in ant community composition across the habitats (one-way ANOSIM, global R = 1.00, *p* = 0.004).

### 3.2. Invader Abundance Drives Decline in Native Richness

Generalized linear mixed models (GLMM) showed a significant decline in native ant diversity in the invaded plots. Native species richness was significantly lower in the *S. invicta*-invaded open habitat (SI) compared to both forest edge (EC) and secondary forest (SF) (*p* < 0.001). The data clearly indicated competitive displacement, with total ant abundance highest in *S. invicta*-invaded open habitat (SI), primarily due to the dominance of *S. invicta*. Conversely, native ant abundance in SI was significantly reduced compared to EC and SF (*p* < 0.001, Table 1). Furthermore, Pearson correlation analysis indicated that the native ant species richness was significantly and negatively correlated with abundance of *S. invicta* foragers (*R* = −0.67, *p* = 0.02). No significant correlation was identified between *S. invicta* nest density and native ant abundance and species richness (*p* > 0.05). This suggests that the primary mechanism of impact is the intense pressure exerted by the large number of foraging individuals, rather than simply the presence of nests.

### 3.3. Functional Traits Distributions Reveal Niche Overlap Change and Competitive Filtering

**Figure 2:**
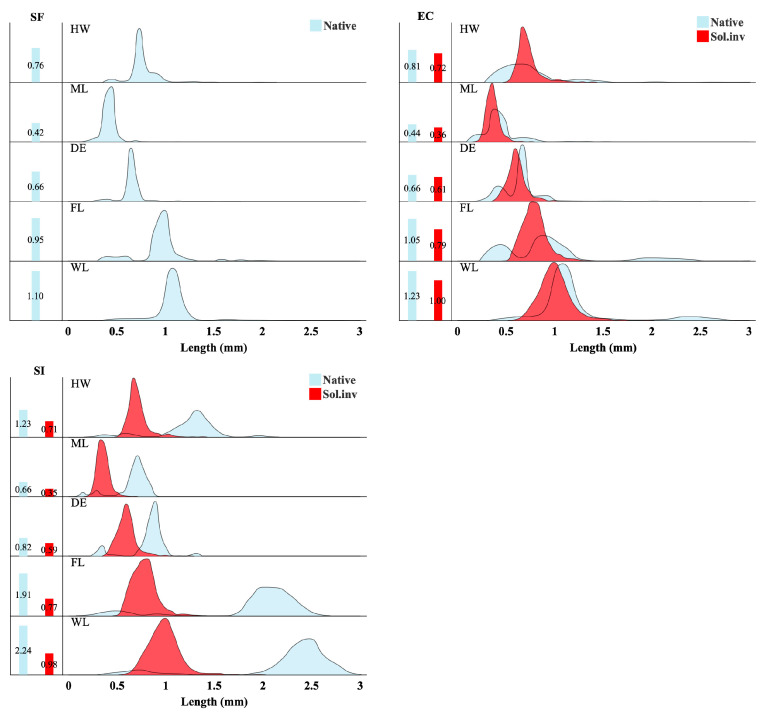
Distribution of functional traits in ant communities across three habitats. Table ridge plots within each panel illustrate the kernel density for native ants (blue) and *S. invicta* (red) across five traits. The *x*-axis shows trait length in millimeters. To the left, bar charts depict the mean values for each group for each trait. The abbreviations are: HW (head width), ML (mandible length), DE (distance between eyes), FL (hind femur length), and WL (Weber’s length).

Kernel density estimation plots, which show the probability distribution of trait values within each community, revealed a competitive exclusion mechanism across habitats (Figure 2). In the secondary forest (SF), trait distributions among native ant species generally overlapped. At the forest edge (EC), the trait distribution of *S. invicta* significantly overlapped with that of native species, especially regarding body size (Weber’s length) and traits related to locomotion (femur length), leading to intense competition. In the *S. invicta*-invaded open habitat (SI), this competition’s outcome was clear: native individuals with traits overlapping *S. invicta* were nearly eliminated. Consequently, the native trait distribution is severely truncated, now dominated by smaller-bodied individuals.

### 3.4. Species-Specific Trait Shifts as Evidence for Trait Displacement

**Figure 3:**
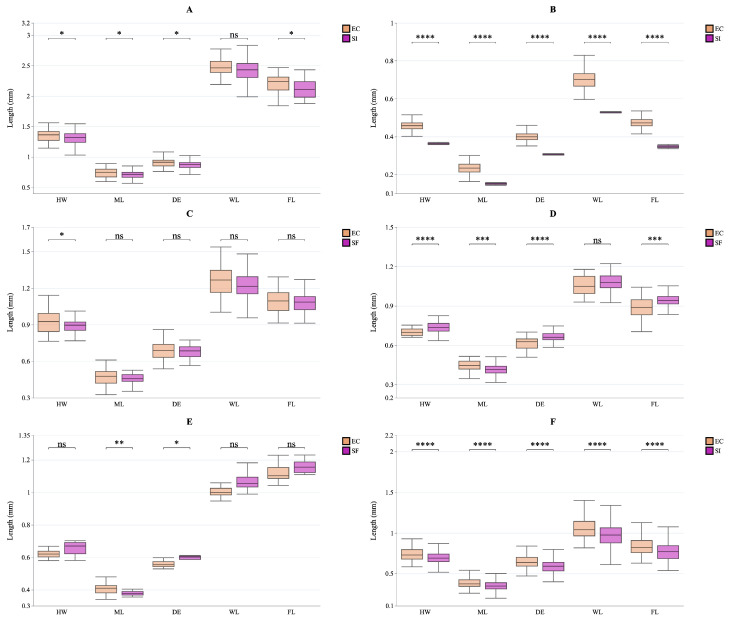
Species-specific shifts in functional traits for key native species across different habitats. Each panel shows a different species with boxplots of five traits, illustrating intraspecific trait variation (ITV) and directional changes across habitats. (**A**) *Camponotus cornis*; (**B**) *Cardiocondyla nuda*; (**C**) *Lasius alienus*; (**D**) *Kartidris sparsipila*; (**E**) *Pheidole spathifera*; (**F**) *S. invicta*. Boxes depict the interquartile range, the line indicates the median, and whiskers extend to 1.5 times the IQR. Asterisks indicate significance levels from *t*-tests comparing trait values between habitats (*: *p* < 0.05; **: *p* < 0.01; ***: *p* < 0.001; ****: *p* < 0.0001; ns: not significant).

We analyzed intraspecific trait variation (ITV) for key native species that co-occur in at least two different habitats (see Figure 3). Our findings revealed highly species-specific morphological changes in response to the presence of *S. invicta*. Specifically, *Camponotus cornis* individuals from the *S. invicta*-invaded open habitat (SI) showed a targeted reduction in HW, ML, DE, and FL, but not WL, compared to those in the forest edge (EC) (ANOVA, *p* < 0.05; see Figure 3A). *Cardiocondyla nuda* showed a clear pattern of miniaturization, with individuals from the *S. invicta*-invaded open habitat (SI) being significantly smaller across all five traits (ANOVA, *p* < 0.001; see Figure 3B). In contrast, comparisons of three native species (*Lasius alienus*, *Kartidris sparsipila*, *Pheidole spathifera*) between the two habitats-forest edge (EC) and secondary forest (SF)-revealed either no significant differences or inconsistent patterns. This suggests that the notable changes observed in *C. cornis* and *C. nuda* are specific responses to the presence of the invader (see Figure 3C–E). We note that some native genera exhibit worker polymorphism with major and minor subcastes; our sampling focused on foraging workers and did not quantify subcaste proportions per colony or habitat, which may influence apparent trait distributions. Future work should explicitly track subcaste composition and task allocation across the invasion gradient. Finally, we examined the invader’s morphology itself and discovered a consistent, highly significant pattern: *S. invicta* individuals from invaded open habitats (SI) were notably smaller than those from forest edges (EC) (see Figure 3F). This size difference was consistent across all five traits measured (ANOVA, all *p* < 0.0001), suggesting that the phenotype of foraging workers is not fixed but changes markedly with habitat type and likely invasion stage.

### 3.5. Hierarchical Analysis Confirms Competitive Filtering as the Dominant Assembly Process

**Figure 4:**
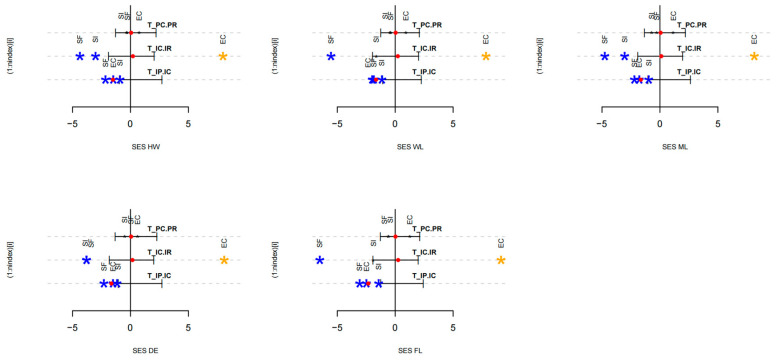
T-statistic analysis of community assembly mechanisms for ants in three habitats. HW: head width, WL: Weber’s length, ML: mandible length, DE: distance between eyes, FL: femur length. Categories include secondary forest (SF), forest edge (EC), and *S. invicta*-invaded open habitat (SI). The figure compares observed values of three key T-statistics against their null model. The horizontal line indicates the 95% confidence interval of the null model, generated from 999 permutations. Observed values (red dots) within this interval are deemed not significant. Asterisks mark values significantly different from the null model (*p* < 0.05): blue for lower, orange for higher.

The T_PC/PR_ index indicates no significant deviation from the null model for any of the five traits across all three habitats. This implies that environmental conditions in this area do not select species based on their average traits. The composition of ant species within each habitat appears to be random. Conversely, the T_IC/IR_ index, which assesses trait variance within communities in relation to the regional pool, shows significant and habitat-specific differences. In the secondary forest (SF), all five traits were notably lower than the null model predictions, pointing to environmental filtering where specific pressures restrict trait variation within a narrow range. Conversely, in the forest edge (EC), all five traits surpass the null model expectations, indicating strong trait divergence likely caused by interspecific competition filtering, which promotes broader trait variability to reduce niche overlap. The results from the *S. invicta*-invaded open habitat (SI) are mixed: HW, ML, and FL are significantly below the null model, reflecting competitive filtering by *S. invicta*, whereas WL and DE show no significant difference, implying a more random assembly for these traits. The T_IP/IC_ index measures trait overlap among coexisting species, revealing a clear pattern: across three habitats and five traits, observed trait values are consistently much lower than the null model predictions. This suggests that species partition their trait space with little overlap, a typical indication of limiting similarity and niche partitioning that promotes coexistence (Figure 4).

## 4. Discussion

Our study provides a comprehensive analysis of how *S. invicta* structures native ant communities across different habitats and seasons by examining changes in taxonomic and functional diversity. A key finding of our research is that the impact of *S. invicta* on native ant communities is highly context-dependent, varying significantly with habitat type and invasion intensity. These findings do not refute the classic theory of niche differentiation; instead, they illuminate how it operates under extreme competitive conditions. We demonstrate that intense competitive pressure from *S. invicta* forces a constrained form of niche differentiation, whereby surviving native species are filtered into a narrow, shared functional space. This process, mediated by intraspecific trait variation, results in a community-level pattern of trait convergence, revealing a crucial mechanism by which invaders can rewrite the rules of community assembly.

Classic theory predicts that native species should become more dissimilar from an invader to reduce competition. Our results support this prediction’s premise. The intense pressure from *S. invicta* acts as a powerful selective filter, creating a vast, occupied zone in the functional landscape that is inhospitable to native species with overlapping traits. However, instead of scattering across a wide range of alternative niches, the surviving native ant community became functionally more clustered. This outcome offers a nuanced alternative to the classic pattern of functional homogenization often reported in invasion literature [8,44,45]. This apparent paradox is resolved when niche differentiation is viewed not as an unconstrained process, but as one limited by the availability of viable niche space. The invader’s dominance appears so overwhelming that it closes off most potential avenues for differentiation, leaving only a very narrow functional zone available [46]. Consequently, all surviving species, in their independent efforts to differentiate from the invader, are channeled into this same limited space. The resulting pattern is one of community-level convergence, not because species are converging on each other, but because they are all being forced away from the invader and into the only viable portion of the niche landscape [7,47]. This finding specifies the conditions under which the fundamental process of niche differentiation leads to a convergent pattern, a crucial insight for predicting community responses to severe biotic pressures.

The rapid community-level reconfiguration was achieved through intraspecific trait variation (ITV). While the importance of ITV in community assembly is increasingly recognized [26,48,49], its role in mediating biotic resistance in animal communities has remained largely unexplored [50]. The significant, directional shifts in the traits of surviving native species like *Cardiocondyla nuda* and *Camponotus cornis* are a clear example of rapid, contemporary adaptation. While phenotypic plasticity represents the most parsimonious mechanism for these observed shifts, the contribution of concurrent rapid microevolution cannot be entirely ruled out. These species persisted not because they were inherently different from the invader, but because their populations contained enough variation for selection to act upon, allowing them to execute the necessary niche shifts into the constrained functional space. The adaptive significance of these morphological shifts is apparent in the context of *S. invicta*’s aggressive, territorial foraging strategy [16]. This result challenges the conclusions of many invasion studies that rely on species-mean traits. Such approaches would have missed this critical dynamic and might have wrongly concluded that the surviving species were simply pre-adapted colonists. Our work empirically demonstrates that ITV is not just statistical noise but the primary engine allowing native communities to respond and reconfigure in ecological, rather than evolutionary, time. This finding contrasts with studies in more stable environments that report limited functional ITV in invertebrate communities [25,26], highlighting the need to incorporate individual-level data into future studies of community assembly and invasion impact.

It is also important to address the role of worker polymorphism in the native species’ responses. Genera such as *Pheidole* and *Camponotus* are known to have polymorphic workers, including distinct major and minor sub-castes. We have been careful in our methodology regarding this: due to their significant morphological differences, major workers of *Pheidole* were entirely excluded from our trait measurements. For *Camponotus*, while a small number of larger individuals that could potentially be major workers may have been included, our analysis is overwhelmingly based on the foraging worker collected in traps. This focus on foragers means our study was not designed to quantify shifts in sub-caste ratios in response to invasion pressure. Such colony-level shifts in task allocation and morphological investment represent a crucial axis of response that warrants dedicated future investigation.

A notable finding is the systematic trait variation exhibited by *S. invicta* itself. We found that workers in heavily invaded core habitats were consistently smaller than those at the invasion edge. This observation can be explained by two primary, non-mutually exclusive explanations. An alternative explanation for smaller foraging workers in core SI sites is colony-level division of labor: colonies may allocate smaller workers to routine surface foraging while larger workers perform defense and nest construction, shifting the observed forager size distribution without requiring genetic change. This observation aligns with ecological theory on density-dependent selection, where intraspecific competition in core populations can select for different life-history strategies compared to expanding, edge populations [51,52]. This intraspecific variation within the invader has important implications. Most invasion studies treat invasive species as phenotypically static, yet our results demonstrate their potential for significant trait plasticity. We propose that a constraint-based explanation rooted in density-dependent phenotypic plasticity is the most parsimonious mechanism driving this size reduction, where intense intraspecific competition for resources and other abiotic factors like temperature constrain worker development. However, we cannot exclude the possibility of concurrent rapid microevolution [51]. This adaptability may contribute to invasion success by allowing invaders to optimize their phenotypes for local conditions [53]. The context-dependent nature of the invader’s traits suggests that invasion impacts are not static but may evolve as the invader population matures, a temporal dimension that warrants greater attention in invasion ecology. In our system, SI sites have been colonized for approximately three to five years, spanning multiple colony life cycles; thus, observed trait shifts likely reflect early-to-mid invasion dynamics driven primarily by density-dependent selection and plasticity, rather than a long-term evolutionary equilibrium.

## 5. Conclusions

Our research demonstrates that the foundational principle of niche differentiation can lead to unexpected patterns under extreme biotic pressure. A dominant invader does not simply cause species loss, but actively reshapes the functional landscape, forcing surviving native species into a narrow, convergent niche space. This process of constrained niche differentiation is not a rejection of classic theory but a critical specification of it. It highlights how the outcomes of competitive interactions are contingent on the intensity of the selective pressures. We identify intraspecific trait variation (ITV) as the key mechanism enabling this rapid community reorganization. This finding establishes a clear mechanistic link from individual-level adaptation to community-level functional patterns. Our work thus provides a more robust and integrative understanding of invasion impacts, which is essential for predicting which communities may harbor the resilience to persist in our rapidly changing world.

## Figures and Tables

**Table 1:** Statistical results of the Poisson distributed generalized linear mixed effect models (GLMM).

Models		Estimate	Std. Error	z	*p*
Species richness	Poisson (Intercept)	2.203	0.176	12.507	<0.001
	Sites-SF	−0.037	0.192	−0.192	0.847
	Sites-SI	−0.916	0.252	−3.632	<0.001
Abundance	Poisson (Intercept)	4.523	0.190	23.833	<0.001
	Sites-SF	0.243	0.056	4.345	<0.001
	Sites-SI	0.385	0.054	7.093	<0.001
Species richness_local ants	Poisson (Intercept)	2.084	0.192	10.83	<0.001
	Sites-SF	0.078	0.198	0.396	0.692
	Sites-SI	−1.119	0.288	−3.887	<0.001
Abundance_local ants	nbinom2 (Intercept)	3.937	0.240	16.437	<0.001
	Sites-SF	0.800	0.112	7.143	<0.001
	Sites-SI	−1.377	0.155	−8.886	<0.001

Note: A negative binomial distribution was chosen for local ant abundance because of overdispersion in the data; all other variables were modeled with a Poisson distribution. These include secondary forest (SF), forest edge (EC), and *S. invicta*-invaded open habitat (SI).

## Data Availability

https://doi.org/10.6084/m9.figshare.29561894.v1 (accessed on 1 October 2025).
